# Interdisciplinary innovative approach to an aggressive ossifying fibroma case: Integrating 3D surgery and customized reconstruction in maxillofacial and dental care

**DOI:** 10.4317/jced.61581

**Published:** 2024-05-01

**Authors:** Íñigo Aragón-Niño, José-Luis del Castillo Pardo-de Vera, Pablo Garrido-Martínez, Pedro-Manuel Losa-Muñoz, Álvaro-Damián Moreiras-Sanchez, José-Luis Cebrián-Carretero

**Affiliations:** 1 Medical Resident. Oral and Maxillofacial Surgery Department. La Paz University Hospital. Madrid, Spain; 2 Physician attending / Faculty. Oral and Maxillofacial Surgery Department. La Paz University Hospital. Madrid, Spain; 3 Dentist. Oral and Maxillofacial Surgery Department. Hospital La Luz. Madrid, Spain; 4 Head of the Department. Oral and Maxillofacial Surgery Department. La Paz University Hospital. Madrid, Spain

## Abstract

Aggressive ossifying fibroma is a benign fibro-osseous disorder characterized by its aggressive behavior, which complicates its management. In this article, we present a case involving the recurrence of this condition in the maxillary region, with orbital and dental involvement, in a patient who had previously undergone surgery and reconstruction with a microvascularized free fibula flap. A multidisciplinary approach involving maxillofacial surgery and dentistry was employed to deliver a customized and entirely satisfactory solution for the patient. The use of 3D surgery was integral to our approach, encompassing pre-surgical digital planning and the transfer of this planning to the operating room via navigation software. Customized surgical cutting guides facilitated precise resection, while a personalized polyether ether ketone (PEEK) prosthesis was utilized for reconstruction of the malar and infraorbital region. Pre-prosthetic computer-aided design/computer-aided manufacturing (CAD/CAM) surgery, along with dental rehabilitation using transepithelial abutments and dental prostheses on a titanium framework, were employed for dental restoration. During the postoperative period, mobility in the reconstructed maxilla was observed due to the loss of support from the initial reconstruction plate. This was addressed by replacing the plate with a custom-made titanium plate, designed to accommodate the location of the transepithelial abutments and prevent disruption of the dental rehabilitation. This case demonstrates the potential of new technologies when applied within the collaborative framework of maxillofacial surgeons and dentists, enabling effective and definitive solutions in complex reconstruction cases.

** Key words:**Aggressive ossifying fibroma; 3D surgery; customized reconstruction; complex dental reconstruction.

## Introduction

Complex cases involving lesions in the craniofacial region often necessitate a multidisciplinary approach involving collaboration between maxillofacial surgeons and dentists to ensure comprehensive and successful patient outcomes. Through combined planning, each professional contributes their expertise and knowledge to develop a cohesive treatment plan wherein excision, reconstruction, and dental rehabilitation seamlessly integrate like pieces of a puzzle.

In addition to the customary teamwork, the application of technology, particularly 3D surgery, offers significant advantages. This includes virtual planning, navigation assistance, the development of precision cutting guides, and the creation of customized prostheses. These technologies, coupled with pre-prosthetic CAD/CAM surgery and tailored dental rehabilitation treatments, enhance the synergistic relationship between the two disciplines. Consequently, they enable the attainment of an unprecedented level of personalized medicine tailored to the individual patient’s needs.

## Case Report

This article presents the case of a 43-year-old male patient diagnosed in June 2018 with a bone fibrous lesion consistent with aggressive ossifying fibroma in the right orbitomalar region. In September 2018, he underwent surgery at another institution where a right maxillectomy was performed, along with placement of an orbital mesh and reconstruction using a microvascularized right fibula flap.

In December 2018, imaging studies revealed the persistence of a lesion in the right sphenoid and middle cranial fossa, which remained stable until November 2019, when growth of the lesion was observed. At this point, he was referred to our service for evaluation and consideration of further resection.

Following evaluation, scheduled surgery was determined to be necessary to remove the lesion, with subsequent reconstruction using a customized orbitomalar polyether ether ketone (PEEK) prosthesis assisted by intraoperative neuronavigation.

Subsequently, two additional procedures were performed: dental rehabilitation involving the placement of implants on guided fibula and replacement of the previous reconstruction plate with a patient-specific instrumentation (PSI) plate.

-Aggressive fibroma ossificans:

Among benign fibro-osseous lesions, fibroma ossificans is noteworthy. Although benign in nature, it is classified as a true neoplasm characterized by an encapsulated proliferation of fibrous tissue. It typically affects the maxillae, particularly the mandible in the molar region (1). The growth of fibroma ossificans is slow and can lead to dental displacement and even root resorption of the teeth.

Diagnosing fibroma ossificans necessitates clinical-radiological correlation with intraoperative findings, as its histology may exhibit similarities with other fibro-osseous lesions (2,3).

-Surgical planning:

Upon referral to our center, various treatment options were evaluated, leading to the decision to proceed with surgical intervention to remove the recurrent area and reconstruct using a patient-specific instrumentation (PSI) prosthesis. For the orbitomalar reconstruction, it was determined that a polyether ether ketone (PEEK) prosthesis would be suiTable due to its exceptional adaptability, biomechanical properties, and ability to integrate with bone tissue (4-7).

**Figure 1:**
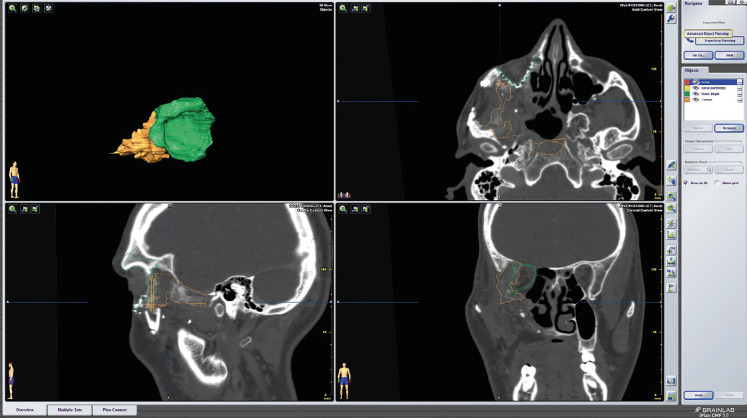
Preoperative planning with Brainlab Elements software.

Opting for a customized solution from the outset was deemed advantageous over traditional alternatives, particularly given the precision afforded by surgical cutting guides. This precision is crucial in addressing a benign yet aggressive pathology where every millimeter of resection is significant. The entire procedure was meticulously planned using specific Craniomaxillofacial (CMF) Planning software provided by Brainlab Elements® (Brainlab AG), with this planning seamlessly transferred to the operating room for intraoperative neuronavigation, (Fig. 1).

-Extirpation and customized reconstructive surgery with dental rehabilitation:

This case report combines three custom reconstructive surgical techniques.

1. A custom orbitomalar PEEK prosthesis for reconstruction of the defect after guided resection with cutting guides.

2. Dental rehabilitation on microvascularized fibula free flap with titanium framework on multi-unit type transepithelial abutments using CAD/CAM planned guided surgery and positioning guide.

3. Replacement of the previous reconstruction plate with a new customized plate of the maxillary segment reconstructed with the fibula flap.

1. Excision of the lesion with surgical guides and reconstruction with a customized PEEK prosthesis:

To facilitate the excision of the recurrent area, a customized cutting guide tailored to the patient’s anatomy was meticulously crafted to precisely delineate the targeted region for resection.

The chosen surgical approach involved a coronal approach with preauricular extension, followed by a guided orbitozygomatic ostectomy to remove the recurrent area.

For the reconstruction of the orbitomalar defect, a customized polyether ether ketone (PEEK) prosthesis (DePuy Shynthes®) was employed to seamlessly cover the previously resected area as per the guidance provided by the cutting guides. This combination of guided resection surgery and customized prosthesis ensures the highest level of adaptability to the patient’s unique anatomy, thereby enhancing the quality of assistance throughout the resection and reconstruction process.

**Figure 2:**
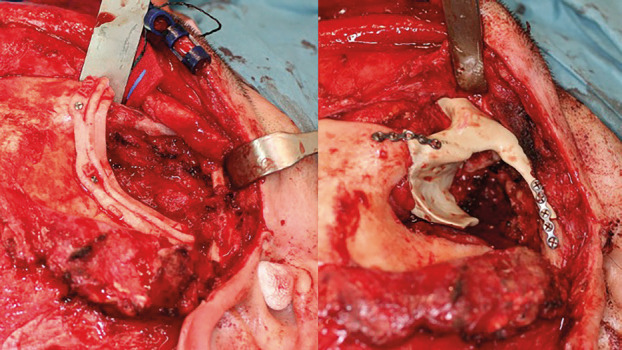
Cutting guide and the PEEK prosthesis.

The PEEK prosthesis was securely affixed using osteosynthesis plates to the healthy bone margins and further supplemented with a titanium mesh to provide additional support in the temporal region, (Fig. 2).

2. Dental rehabilitation on microvascularized fibula free flap:

The reconstruction process is completed with a functional and aesthetically pleasing dental restoration, which involves CAD/CAM preprosthetic surgery for the creation of a guide for implant placement at the sites of teeth 11, 13, 15, and 17.

The utilization of CAD/CAM technology in preprosthetic surgery has been shown to offer noTable advantages in both the surgical procedure and the subsequent adaptation of implants (8,9).

For dental rehabilitation, four Multiunit type transepithelial abutments (Ticare®) are employed alongside 3.75 x 10 mm osseointegrated implants. These abutments are then attached to a milled cobalt-chromium structure model BioCam (Ticare®), which serves as the foundation for the definitive dental prosthesis.

**Figure 3:**
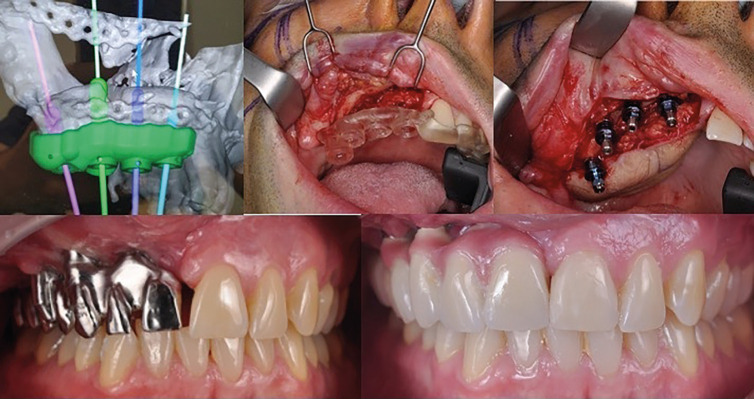
Step-by-step dental rehabilitation.

In this particular case, it was decided to leave the surgical area of the dental arch free to facilitate improved hygiene and to allow for easier follow-up examinations of the patient, (Fig. 3).

3. Replacement of the previous reconstruction plate with a new custom plate:

Postoperatively, the patient exhibited mobility of the maxillary segment reconstructed with the fibula flap. Consequently, it was deemed necessary to replace the reconstruction plate from the previous surgery with a new customized reconstruction plate.

**Figure 4:**
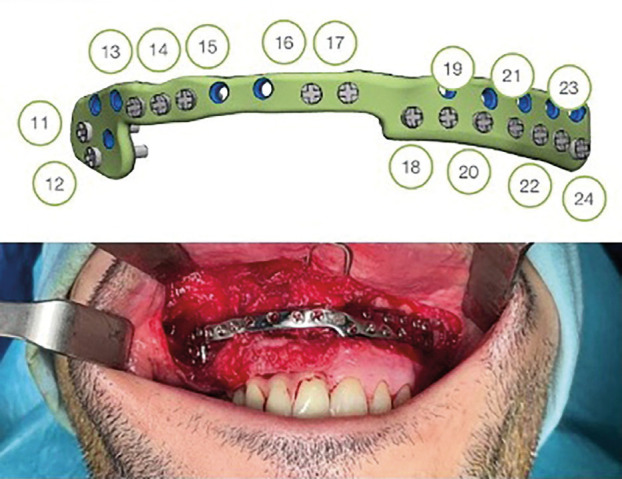
Planning and placement of a customized reconstruction plate with preservation of the dental rehabilitation.

The design of this new plate (Customimplants®) took into consideration not only the positioning of the screws from the previous reconstruction plate but also the placement of the implants. This ensured that during the surgery, the plate could be replaced without the need to remove the implants. This approach preserved the dental structure and upheld the integrity of the dental rehabilitation throughout the procedure, (Fig. 4).

This case serves as a compelling example of how advancements in technology enable closer collaboration between maxillofacial surgeons and dentists, ultimately leading to more seamless and effective patient care.

## Discussion

A customized solution was proposed to address the recurrence of the lesion while preserving the previous reconstruction of the fibular microsurgical flap, all with minimal aesthetic impact and ensuring functional dental reconstruction. The key to achieving the best surgical outcome lies in optimal surgical planning. In recent years, the concept of 3D surgery has been developed, allowing for pre-surgical planning, virtual design of incision locations, and desired outcomes before surgery (4-6,10). This is particularly useful in the field of maxillofacial surgery reconstructive surgery and in dental surgery for dental rehabilitation (11).

3D surgery enables us to transition from on-site surgery planning to pre-surgical planning of desired movement, with simulation and visualization of the result at the bone and soft tissue levels. Surgical guides allow us to transfer information from virtual planning to the operating room. The use of 3D surgery has shown improvement in surgical outcomes, including precision in resection, lower reintervention rates, shorter surgical times with less comorbidity, and improved patient satisfaction post-treatment (4-6,12).

In this case, the condition is benign but with aggressive behavior, so every millimeter of resection is crucial to ensure a balance between curative surgery and optimal aesthetic and functional outcomes for the patient. The combination of reconstructive head and neck surgery performed by Maxillofacial Surgery with pre-prosthetic surgery and subsequent customized dental restoration by Dentistry achieves excellent aesthetic and functional results, as well as high patient satisfaction.

3D surgery allows for the creation of personalized models and is already providing significant advantages in all phases of treatment: diagnostic, therapeutic, and follow-up, bringing our clinical practice closer to the highest standards of personalized medicine.

## Data Availability

The datasets used and/or analyzed during the current study are available from the corresponding author.
